# Action–effect knowledge transfers to similar effect stimuli

**DOI:** 10.1007/s00426-023-01800-4

**Published:** 2023-02-23

**Authors:** Sarah Esser, Hilde Haider, Clarissa Lustig, Takumi Tanaka, Kanji Tanaka

**Affiliations:** 1 Department of Cognitive Psychology, University of Cologne, Cologne, Germany; 2 Graduate School of Humanities and Sociology and Faculty of Letters, The University of Tokyo, Tokyo, Japan; 3 Faculty of Arts and Science, Kyushu University, Fukuoka, Japan

## Abstract

The ability to anticipate the sensory consequences of our actions (i.e., action–effects) is known to be important for intentional action initiation and control. Learned action–effects can select the responses that previously have been associated with them. What has been largely unexplored is how learned action–effect associations can aid action selection for effects that have not previously associated with an action but are similar to learned effects. In two studies, we aimed to show that when presented new, unknown action–effects, participants select the responses that have previously been associated with similar action–effects. In the first study (*n* = 27), action–effect similarity was operationalized via stimuli belonging to the same or different categories as the previously learned action–effects. In the second study (*n* = 31), action–effect similarity was realized via stimuli that require comparable motor responses in real life. Participants first learned that specific responses are followed by specific visual effect stimuli. In the test phase, learned effect stimuli, new but similar effect stimuli and new but dissimilar effect stimuli were presented ahead of the response. The findings revealed that both learned effect stimuli and new similar effect stimuli affected response times, whereas new dissimilar effects did not. When a learned or a new similar effect was followed by a learned response, compared to an unlearned response, the responses were faster. We interpret these findings in terms of action–effect learning. The action–effect once bound to an action is used to select an action if a similar effect for which no action has been learned yet is presented. However, it is noteworthy that, due to our design, other explanations for the found transfer are conceivable. We address these limitations in the General Discussion.

In everyday life, it might happen that we intend to do something we have done never before. The theoretical problem in such a case is that, in principle, the search space for the action most likely to achieve the desired outcome is unlimited. So, how can we reduce the search space to find and activate a potential action in time which might enable us to achieve the intended goal. A simple, but probably efficient solution is to look for already known action–effects that are similar to the intended goal and to conduct the action that had been associated with this effect in the past. More generally, this means, we try to find a similar action–effect (or goal) for which we know already how to produce it. On the one hand, this narrows the search space for promising actions and, on the other hand, explains how we can translate a new action goal into an action. The current study aimed to provide first preliminary empirical evidence for this consideration.

A large body of research shows that humans are able to learn the various sensory consequences (i.e., effects) that follow our actions (Shin et al., 2010, for a review). For example, pressing keys on the keyboard leads to certain clicking sounds, proprioceptive and haptic feedback, and letters appearing on the screen. Learning about such sensory consequences of actions is assumed to have an important role in selecting and initiating the respective actions, with different theoretical conceptions on how the anticipation of a known action–effect relation can influence offline and online control of actions (Hommel, 2019; Hommel et al., 2001; Ziessler et al., 2004).

In typical experimental settings that investigate action–effect anticipation, participants first learn that certain stimuli follow specific responses. For this purpose, they respond to a stimulus which is then followed by a particular effect. In the following phase, participants will then perceive or anticipate the learned effect and have to respond with either the associated response or a different one. This usually leads to fast reaction times when the same response is required and slower reaction times, when a different response is required (Elsner & Hommel, 2001). This has not only been shown for effect stimuli being externally present ahead of the response, but also for internal anticipations of these effects without any direct presentation. For instance, Kunde et al. (2002) instructed the participants to respond with either a strong or a soft keypress followed by a loud or soft effect-tone. The results showed faster responses when the effect-tone was compatible to the action than when it was incompatible.

These findings can be explained via bi-directional associative contingency learning (Elsner & Hommel, 2004). Once a specific action has contingently led to one or more specific sensory consequences, the respective motor commands and the internal sensory representations become associated. Due to the assumption that this association is bi-directional, any activation of the sensory codes, either externally by perceiving the sensory events or internally by anticipating these events, leads to the activation and possibly execution of the corresponding motor commands. Historically, this general concept is traced back to James who called it the *Ideomotor Principle* (1890). This term is still commonly used nowadays for models of voluntary action control where the internal anticipation of sensory consequences to initiate motor commands has become an important concept that can further be found in relation to sense of agency (Moore & Obhi, 2012), where the anticipation of effects is related to the experience of causing them and the development of mirror neurons (Heyes, 2010), where perceiving the actions of others or rather the sensory consequences of these actions elicit corresponding motor activations in oneself.

While numerous studies have shown the importance of action–effect learning over various paradigms, in everyday situations, there is variety in both, the sensory outcomes of an action (e.g., every keyboard sounds and feels different) as well as the responses that are required to achieve these effects (e.g., different spaces between the different keys). In some situations, we may even be uncertain about which precise action might lead to a desired outcome. For example, when we learn a new language, there might be sounds that are somewhat similar or even totally different from the sounds that we have learned to pronounce so far. Thus, the question arises how acquired knowledge about action–effect relations can help to select the action that is most likely to achieve a desired outcome which only has some resemblance with the outcomes we have learned to achieve.

Ziessler et al., (2004) proposed a model of action–effect learning based on sensory-motor feedback and feedforward loops that can help to explain how the anticipation of similar, yet unlearned effects can be translated into actions. Their model combines the basic idea of associative action–effect learning with predictive models of sensory-motor control (Wolpert & Kawato, 1998). The integration of associative action–effect learning and predictive models has been elaborated further by Kilner et al., (2015; see also: Adams et al., 2013). Generally, motor responses lead to sensory consequences. Once learned, these sensory consequences can be predicted and compared to the actual sensory outcomes of the action. The resulting prediction error can be used to update and improve future effect predictions. This part is called the feedforward part of the model. Critically, the model also contains a so-called inverse model. This part serves to explain how the anticipation of action–effects can be used to initiate a response. Within the inverse model, a desired effect, a specific goal that should be achieved, is internally represented. The desired effect representation activates learned motor representations that could achieve the effect. Based on these pre-selected motor commands, the forward model can make predictions what the sensory effects of these actions would be. Now, the predicted effects can be compared to the desired effects and, in case of both matching, the action can be executed. If there is no match between desired and predicted effect, the system can use the prediction error to make adjustments to the motor commands. Under the circumstance that the desired effect is something that we have never achieved before, like pronouncing a word in a foreign language, it might not be possible to select the exact, yet unknown motor commands. Nevertheless, there can still be a comparison to similar effects (e.g., similar phonetic sounds) that we have learned already. This will obviously result in some prediction error between the desired and the anticipated effect. That prediction error can be used to make motor adjustments that are most likely to result in an effect similar to the desired one. Once this motor program has been selected and executed, an actual sensory outcome will result. The result will probably differ from what has been anticipated in some way and also from what has been desired. However, the system now can make an advance using the prediction error between the anticipated and the actual effect to produce a slightly different response next time. This way, the actual effects can get incrementally closer to the desired effect.

Thus, predictive models of action–effect learning can be used to make assumptions about how humans are able to select actions that produce new, unknown effects that are similar to already learned effects, that is for which one knows the action–effect relation. Our study will focus on the most basic assumption that perceiving new effects that are similar to already learned effects will select the action that is associated with an already learned effect because this response is so far the best candidate to achieve this new, similar effect. To our knowledge, no study has directly investigated the effect of presenting new but similar effects to test whether their perception elicits the same or a similar response as the already learned effect.

There are, of course, studies that offer an empirical basis. First, there is a large number of studies that investigated the compatibility between intended action–effects and stimuli in the environment (Hommel, 1993; Kornblum et al., 1990). Whenever a stimulus has some overlap with the intended action goal or effect (e.g., an irrelevant stimulus occurring on the left has a beneficial effect on pressing a button to the left), the response to achieve this effect is accelerated. Thus, similarity between perceived stimuli and anticipated or intended effects affect performance. However, it remains to be shown that perceiving stimuli that are similar to learned effects trigger the same or similar responses as the learned effect, when the learned effect is not intended. Some research has been done already to test whether exemplars as learned effects can generalize to their superordinate category (Eichfelder et al., 2022; Földes et al., 2018; Hommel et al., 2003; Koch et al., 2021). However, the evidence for such conceptual generalization is mixed. While Hommel et al. (2003) reported evidence for such a conceptually generalization, Eichfelder et al. (2022) did not and Koch et al. (2021) did find the effect only when the response features are perceptual related to the effect features.

Another interesting finding came from Hazeltine et al., (2002). Participants learned a sequence of motor responses, for which each response was associated with a specific effect-tone. The effect-tone was either response-location specific or effector specific. Whether they could transfer their knowledge to new locations or effectors was dependent on whether they learned that the effect was effector or location specific. The participants seemed to have acquired goal-based sequence representations. This shows that action–effect learning is not merely a rigid link between one specific response and one specific effect, but that anticipating a certain effect can flexibly recruit abstract motor representations that can achieve this effect. In the following study, instead of keeping the effect constant and varying the action, we will take a closer look at the other case which also is important coordinating everyday activities adaptively: selecting a learned action for new effects that are similar to learned effects.

It is important to note that the meaning of “similarity” of action–effects can vary broadly across modalities and complexity in everyday-life situations. In the previous foreign language learning example similar, new auditive effects are to be achieved; in other situations, similarity can mean to achieve visually similar effects, for example when trying to imitate a new dance move. As goals become more abstract, similarity can also encompass more complex effect representations like trying to create an abstract painting or writing a novel. Thus, to incrementally achieve insight into the mechanisms by which new, unlearned goal repetitions can profit from formerly learned action–effect associations, various “effect similarity” should be operationalized in different levels of abstraction. A closer examination of different levels of similarity can, for example, be found in Kornblum et al. (1990) and a theoretical perspective on how action goals shape the perception of new effects that are similar to already learned effects can be found in Prinz (2018). For our two experiments, we used visual stimuli and two different operationalizations of “effect similarity”. In our first experiment, we use stimuli that belong to the same category as the originally learned effect. In our second experiment, we used stimuli that require similar ways of motor interaction.

## Materials and methods: Experiment 1

### Training

**Fig. 1:**
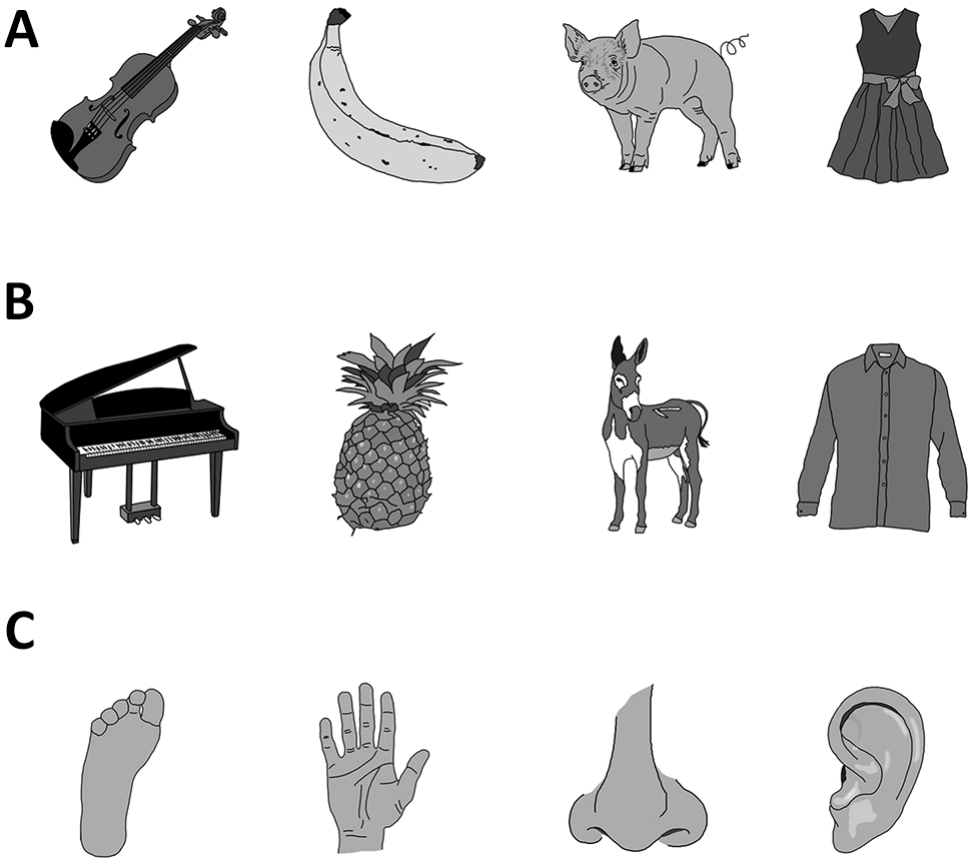
Stimuli used in Experiment 1 during training (**a**) and test (**a**-**c**). During the test, the training stimuli were presented, as well as new similar (**b**) and new dissimilar (**c**) stimuli

**Fig. 2:**
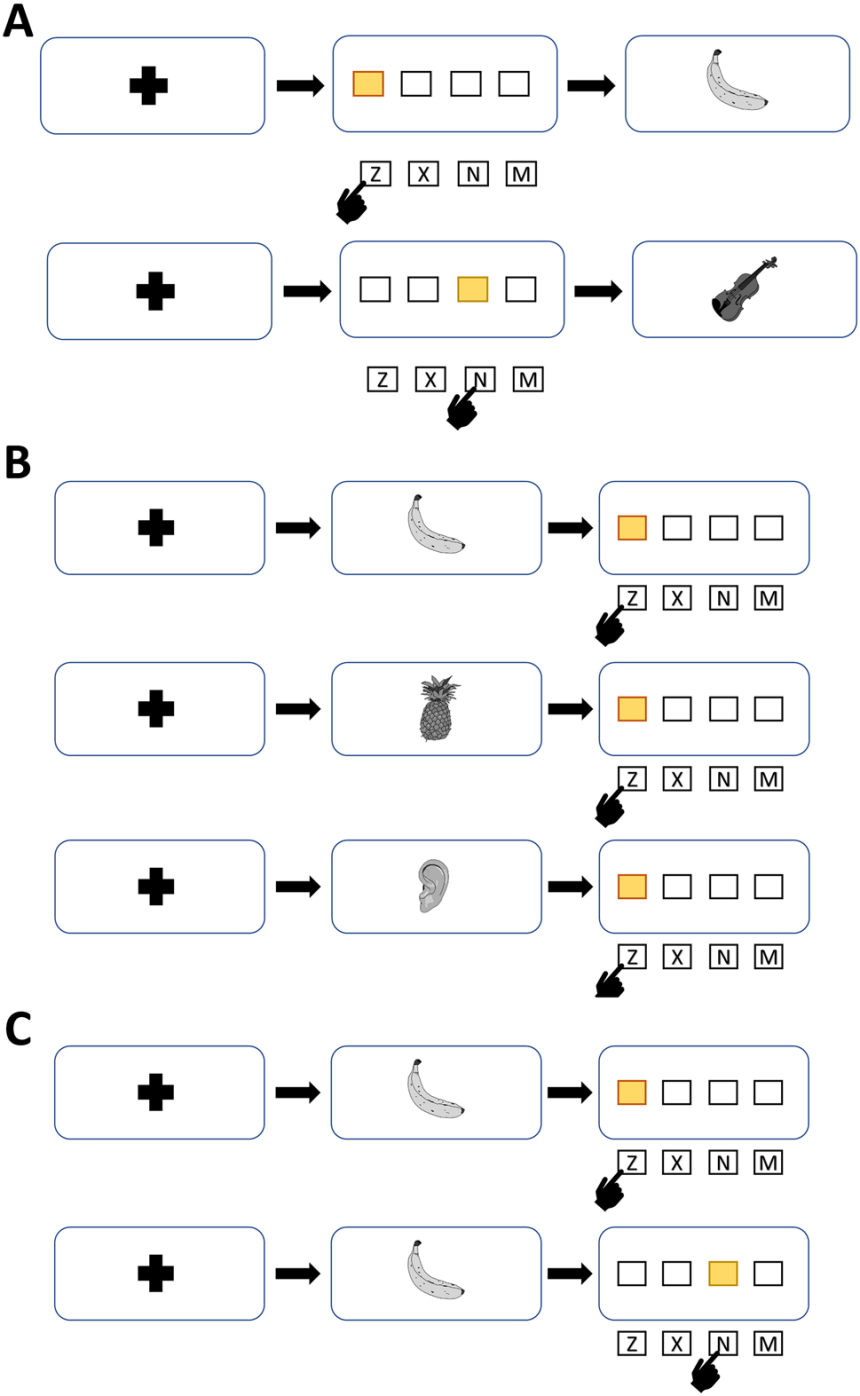
Schematic depiction of the training (**a**) and test task (**b**, **c**). During the test, the responses were preceded by either the same, a similar or a dissimilar stimulus (**b**). The stimuli were followed by the response that has been associated with the respective effect stimulus during training or by a different response (**c**)

In our study, participants were first trained with simple action–effect associations. At the beginning of each trial, participants saw a fixation cross for 1500 ms. 400 ms after the cross disappeared, four horizontally aligned rectangles (100*100 pixels) were shown in the middle of the screen for 800 ms. On each trial, one of the rectangles was highlighted and the participants had to press one of four keys. These four keys were the “z”, “x”, “n”, and “m” keys on a QWERTY keyboard. The keys were spatially mapped to the four rectangles. If the leftmost rectangle was highlighted, “z” had to be pressed, if the rightmost stimulus was highlighted, “m” had to be pressed, and so on. Participants were instructed to respond as fast and as accurately as possible. Immediately after each keypress, a picture of either a pig, a banana, a violin or a dress was shown. Each of these stimuli was 100% contingent with the previously required response location (e.g., “z” was always followed by a banana). The contingent stimulus was shown regardless of the correctness of the participant’s response. All four pictures were taken from the MultiPic Database (Duñabeitia et al., 2018), presented in greyscale and resized to 400*400 pixels. All stimuli are shown in Fig. 1. After 400 ms, the picture disappeared and the fixation cross signaled the start of the next trial. Thus, a trial consists of the spatial response to the four rectangles, the subsequent picture, and the fixation cross. Participants were given 120 training trials in total. Hence, each of the four action–effect associations was presented 30 times. The order of training trials was randomized with the only exception that no two subsequent trials were allowed to be the same. A schematic depiction of the training and the subsequent test phase is presented in Fig. 2.

### Test task

In the following test, the display times and sizing of all stimuli were the same as during training. Importantly, the order of the effect stimuli and the required responses was turned around. Each trial started with a fixation cross. After that, the participants first saw a picture for 400 ms that was either one of the stimuli they have encountered during training or a new one. These new stimuli were either similar to the training stimuli in regard to the category or dissimilar. The similar stimuli were a pineapple, a shirt, a piano, and a donkey. The dissimilar effects were body parts. After the disappearance of the picture, the four rectangles were shown, with one of them being highlighted again. The highlighted position was either the same as the position that has previously been associated with that respective picture during training (e.g., after a banana, “z” had to be pressed) or an unlearned position (e.g., “x”, “n”, or “m", after a banana). Thus, if the picture shown before the response-location task was learned as an action–effect during training, we expect faster reaction times, when the effect was presented before the associated response locations and slower reaction times if an unlearned position was required after a learned effect stimulus (Elsner & Hommel, 2001).

Crucial to the purpose of this study, we likewise expected that pictures that were similar to the learned effects would also be associated with faster response times, if they were followed by a response location that was associated with the similar, learned effect. For example, if a pineapple was shown instead of a banana and the “z” key had to be pressed subsequently, this reaction time should be faster than pressing “x”, “n” or “m” after a pineapple. As a control condition, seeing a dissimilar stimulus (i.e., one of the body-part pictures) would not lead to any increase or decrease of reaction times, regardless of the following response location.

In total, the test task consisted of 144 trials. This means that each singular effect (4 old, 4 similar, and 4 dissimilar effects) was shown 12 times. Within these 12 times, each effect was followed 3 times by the learned response location and 3 times by each of the other 3 unlearned response locations. Of course, the term “learned or unlearned” is by definition only true for the old effects (e.g., if the banana is followed by a “z” response vs. any of the other three responses). For similar effects (e.g., pineapple) it can be applied, if our hypothesis is true. For new dissimilar effects, the terminology of “learned vs. unlearned” is not really applicable as no response location should be associated in any way with these pictures. Thus, within our design, no stimulus–response location association can be acquired, as any picture is followed by each of the four possible response locations in equally.

### Sample

We collected our data online via Psychopy (version 2020.2) and uploaded as JavaScript in Pavlovia (https://pavlovia.org/). Participants were recruited via the online platform Prolific Academic (2022, https://www.prolific.co). They received a payment of 4 £. The only restrictions for participation were that the person had to be from Great Britain or USA and used a QWERTY keyboard. We collected the data of 27 participants. Sample size was based on a G*Power 3.1 analysis (Faul et al., 2007). We expected medium main effect sizes of *η*_*p*_^*2*^ = 0.08, alpha = 0.05, and 1-β = 0.95.^1^ All participants were at least 18 years or older (mean age = 32.4, *SD* = 11.0; 14 women) and gave informed consent before the start of the experiment.

Our exclusion criterion for too many erroneous trials would have been 15% during training or test. One person exceeded this criterion, with 75% errors during training. After screening of the data, we noticed that two participants showed unusually slow responses, both in training and test (> 2.5 SD slower), even after the exclusion of singular RT outliers. Thus, we excluded all 3 participants and the following analyses are based on the remaining 24 participants.

## Results

We analyzed both reaction time (RT) and error data. For the RT analysis, we removed all erroneous trials. We also removed any trial that followed an error due to post-error slowing and trials in which the participants did not react within 1,500 ms. For the learning phase, we only calculated the mean RT and mean error rate for the entire training. For the test task, we computed mean RTs separately for each participant, for each effect type and each response location. During training, the participants responded with a mean RT of 444.28 ms (*SD* = 57.14) and a mean error rate of 2.29% (SD = 2.59%).

For the test data, we computed two separate ANOVAs, one for error rates and one for RTs as dependent variables. In both, the independent variables were *Effect Type* (old, similar, dissimilar) and *Response Location* (learned vs. unlearned). Both factors were treated as repeated within-subject factors.

**Fig. 3:**
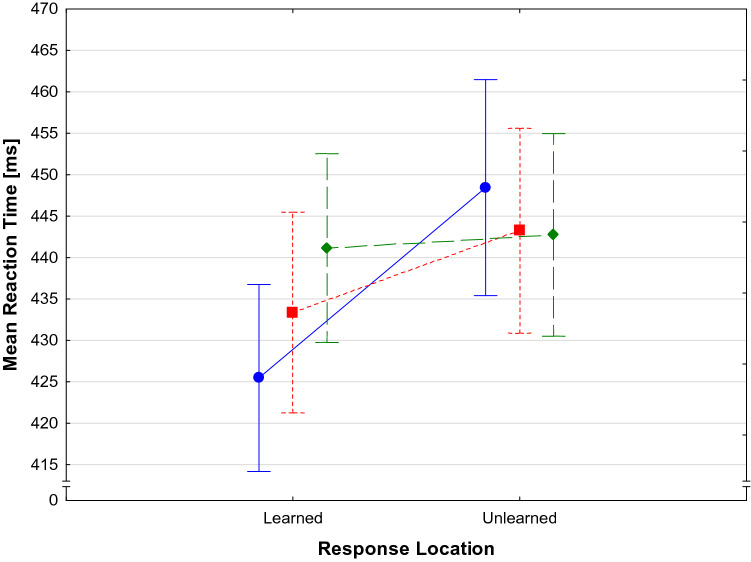
Mean RT during the test task for learned and unlearned response locations for old effects from the training phase (blue, circle, solid line), new but similar effects (red, square, short dashes) and new, dissimilar effects (green, diamond, long dashes). Bars denote standard errors

Mean error rates are shown in Table 1. The ANOVA for error rates yielded no significant main effects and no significant interaction (*F*(2,46) < 1 for Effect Type; F(1,23) < 1 for Response Location, and *F*(2,46) = 1.13, *p* = 0.33 for the interaction). Table 1 also shows the mean RTs separately for the three Effect Types and the learned versus unlearned Response Locations. The ANOVA for RT revealed no significant main effect for Effect Type (*F*(2,46) = 1.81, *p* = 0.180), but a significant main effect for Response Location (*F*(1,23) = 7.74, *p* = 0.011, *η*_*p*_^*2*^ = 0.25). Generally, learned response locations were associated with faster RT (*M* = 433.3 ms, *SD* = 56.83 ms) than unlearned locations (*M* = 444.8 ms, *SD* = 61.14 ms). Most importantly, there was also a significant Effect Type by Response Location interaction (*F*(2, 46) = 4.14, *p* = 0.022, *η*_*p*_^*2*^ = 0.15). Planned contrasts revealed that learned response locations led to faster RT for old effects (*t* (28) = 2.67, *p* = 0.014) as well as for new, similar effects (*t* (28) = 2.19, *p* = 0.039). There was no RT difference for new, dissimilar effects between the different response locations (*t*(28) = 0.44, *p* > 0.6). The RT differences for the effect types and response locations can be seen in Fig. 3.

**Table 1:** Mean error rates and Mean RT for the three Effect Types and learned vs. unlearned Response Locations

Effect type	Response Location		Response Location	
	Learned	Unlearned	Learned	Unlearned
	Error rates		Response times	
Old	0.026 (0.038)	0.045 (0.038)	425.45 (11.29)	448.44 (13.04)
Similar	0.035 (0.038)	0.028 (0.038)	433.36 (12.12)	443.24 (12.35)
Dissimilar	0.026 (0.038)	0.031 (0.038)	441.14 (11.39)	442.73 (12.23)

In brackets, the standard deviations are presented.

## Materials and methods: Experiment 2

The data of this first experiment are in line with our hypothesis. The RT to new stimuli that belonged to the same category as the training effect was faster for response locations that were associated with a similar effect during training. No differences in RT could be found for new, dissimilar effects.

To demonstrate the viability of a mechanism that selects actions on the basis of a predictive mechanism that determines which actions are most likely to achieve a new, desired effect, more operationalizations of “similarity” are needed. In a second experiment, similarity was defined via similar responses in terms of motor commands without the effects belonging to the same object category (e.g., a pushcart and a wheelchair). Additionally, this time, we also used a questionnaire to assess the perceived similarity of the effect pictures.

## Training and test task

**Fig. 4:**
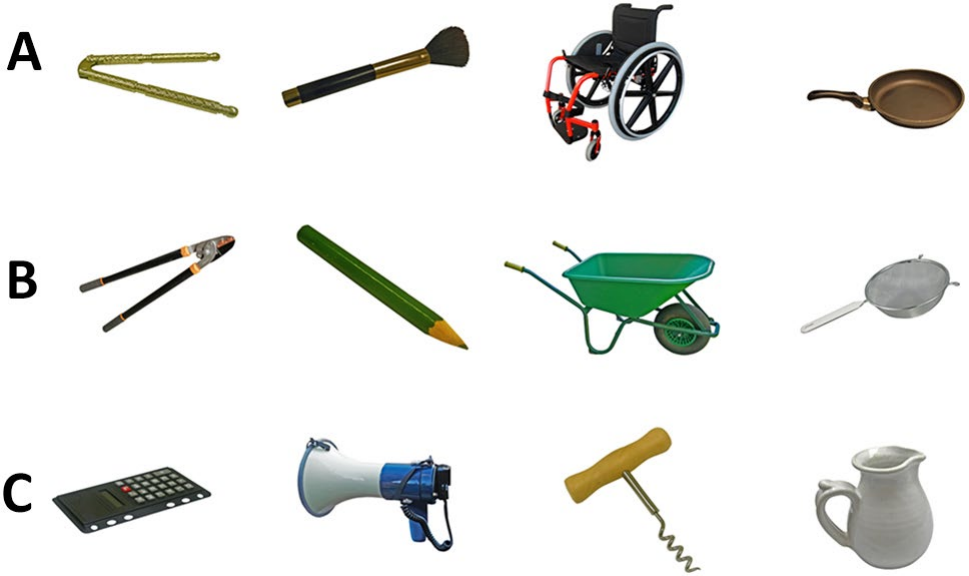
Stimuli used in Experiment 2 during training (**a**) and test (**a**–**c**). During the test, the training stimuli were presented, as well as new similar (**b**) and new dissimilar (**c**) stimuli

The design of both the training and the test task was exactly like that in Experiment 1. The only difference were the stimuli that were used as effects. These were taken from the BOSS database (Brodeur et al., 2010) and are shown in Fig. 4.

## Questionnaire

The questionnaire was created with SoSci Survey (Leiner, 2022) administered to 23 participants after either the participation in the main studies (N = 11) or after a different study (N = 12). Participants were shown eight pairs of stimuli in random order. Each pair showed one of the four stimuli that were used as effects during training. The other stimulus was either one of the four similar stimuli or one of the four new, dissimilar stimuli. Ratings were given on a visual scale of which the endpoints were labeled “dissimilar” and “similar”. Starting from a neutral mid-point on each trial, the participants were asked to move the cursor of the scale to the left or to the right, to a point that they felt was appropriate to describe the similarity of both stimuli. Similarity was not further described to the participants and left to their own interpretation. The participants could not see any numbers on the scale, but in the background, maximal dissimilarity was coded as “0”, “50” described the neutral point and “100” described maximal similarity.

## Sample

Sample recruitment and compensation were the same as in Experiment 1. Based on the G*Power analysis of Experiment 1, we collected the data of 31 participants (mean age = 41.16, *SD* = 11.84). Again, we excluded any participants that had more than 15% errors. This led to the exclusion of two participants. The following analyses of Experiment 2 are based on the remaining 29 participants.

For our questionnaire about the perceived stimulus similarity, we only asked 11 participants from this sample to fill out the questionnaire. We additionally asked 12 participants that took part in a different study to fill out our questionnaire. This way, we were able to see whether participating in the task affects the perceived effect similarity.

## Results

### Questionnaire

We computed an ANOVA with *Effect Type* (similar vs. dissimilar) as a within-subject factor and *Participant Group* (same vs. different study) as a between-subject factor. The subjectively perceived similarity was the dependent variable.

A main effect for Effect Type confirmed that the effect stimuli which we chose as similar were indeed perceived as being more similar to the training effect stimuli than the stimuli that we chose to be dissimilar (*F*(1, 21) = 71.28,* p* < 0.001, *η*_*p*_^*2*^ = 0.772). The mean similarity rating for similar effects was *M* = 64.69 (*SD* = 22.82) and the mean rating for dissimilar effects was *M* = 23.11 (*SD* = 30.46). There was no main effect for Participant Group (*F*(1,21) = 1.73, *p* = 0.202) and no significant interaction (*F* < 1), showing that training with the respective stimuli did not seem to have altered the perception of similarity.

### Training and test task

All analyses were the same as for Experiment 1. During the training task, the mean RT was 464.70 ms (*SD* = 75.16) and the mean error rate was 2.24% (*SD* = 1.90%).

As for the test data of Experiment 1, we computed two ANOVAs; one for error rates and one for RT. The repeated within-subject factors were again *Effect Type* (old, similar, dissimilar) and *Response Location* (learned vs. unlearned).

Concerning the error rate, there was neither any significant main effect nor a significant interaction (*F*(2*,* 56) = 1.60, *p* = 0.212, for Effect Type, *F*(1,28) < 1, for Response Location, and *F*(2, 56) < 1 for the interaction). The mean error rates are shown in Table 2. This table also presents the mean RTs.

**Table 2:** Error rates and mean RTs as a function of Effect Type and Response Location in Experiment 2

Effect type	Response Location		Response Location	
	Learned	Unlearned	Learned	Unlearned
	Error rates		Reaction times	
Old	0.019 (0.031)	0.029 (0.040)	445.29 (13.27)	475.73 (13.78)
Similar	0.013 (0.023)	0.017 (0.036)	459.99 (13.76)	473.75 (14.36)
Dissimilar	0.024 (0.034)	0.026 (0.045)	469.70 (14.52)	473.80 (14.52)

In brackets, the standard deviations are presented.

**Fig. 5:**
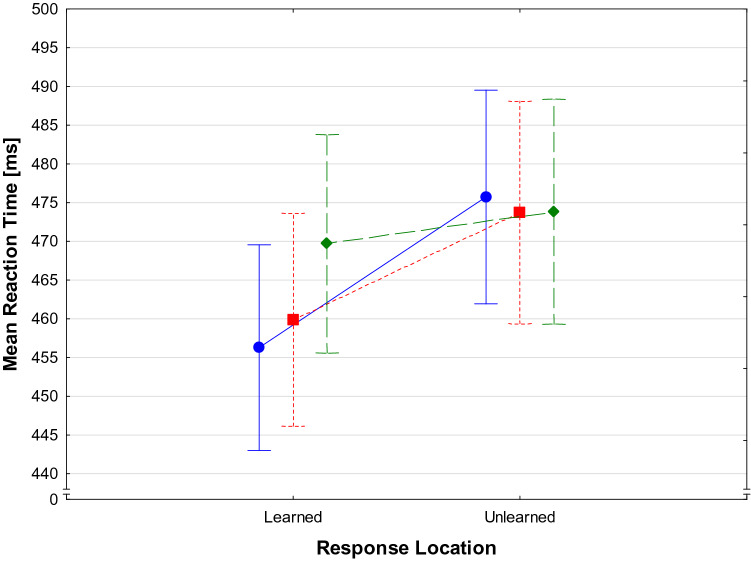
Mean RT during the test task for learned and unlearned response locations for old effects from the training (blue, circle, solid line), new similar effects (red, square, short dashes) and new, dissimilar effects (green, diamond, long dashes). Bars denote standard errors

For RT as dependent variable, there was no main effect for Effect Type (*F*(2,56) = 2.12, *p* = 0.130). The main effect for Response Location was significant (*F*(1,28) = 13.06, *p* = 0.001, *η*_*p*_^*2*^ = 0.318). Like in Experiment 1, learned response locations were associated with faster RT (*M* = 461.96 ms, *SD* = 89.97 ms) than unlearned locations (*M* = 467.35 ms, *SD* = 91.93 ms). Again, most important to our hypothesis was the interaction between Effect Type and Response Location, which, in this case, narrowly missed level of significance (*F*(2, 56) = 2.95, *p* = 0.060, *η*_*p*_^*2*^ = 0.10). As this interaction was very close to significance, we computed the planned contrasts for all effect types. These data showed that, as expected, learned response locations led to shorter RT for old effects (*t*(28) = 3.07, *p* < 0.01). Likewise, there was a significant difference between learned and unlearned response locations for new, similar effects (*t*(28) = 4.03, *p* < 0.001). There was no RT difference for new, dissimilar effects between the different response locations (*t*(28) = 0.82, *p* > 0.41). The RT differences for the effect types and response locations can be seen in Fig. 5.

## Summary and discussion

The data of our two experiments were in line with our hypothesis. Both studies showed the often replicated finding that stimuli that were used as action–effects can lead to a decrease in RT when these effects are presented ahead of the response. What has been less investigated so far and with ambiguous results (Eichfelder et al., 2022; Földes et al, 2018; Hommel et al., 2003; Koch et al., 2021), is whether action–effect knowledge will transfer to conceptually similar effects that have not been associated with any response. In both of our studies, we showed that RTs were moderated by the perceived similarity of new stimuli to the learned effect stimuli. If a response was preceded by a stimulus similar to a learned effect of this response, RTs were faster than the same stimulus followed by a different response. There was no such effect on RT for stimuli that were dissimilar to the learned effects. This was true for two different operationalizations of effect similarity: similarity based on belonging to the same category (Experiment 1) and similarity based on requiring similar motor actions (Experiment 2).

Thus, the findings suggest that an unknown effect selects the response that is most likely to achieve this effect, based on previous experiences with similar effects. We have suggested that this fits well with predictive models of action–effect learning. Anticipating an effect will preselect actions that are able to achieve this effect. An internal prediction of the effects of these actions, determines which action will be selected. Anticipating a new, yet unknown effect should likewise select actions that are most likely to achieve this effect, which should be actions that have achieved comparable effects in the past.

However, our study is not suited to decide whether the internal prediction of effects leads to the selection of that action that produces the smallest prediction error or whether the participants relied on more abstract action–effect knowledge and searched for similar action–effect associations, before then the according action is selected. Thus, probably a simpler associative theoretical approach to action–effect learning can account for these results just as well. Stimulus generalization is an important mechanism that has been considered highly relevant for different learning mechanisms like classical and operant conditioning for many decades (Pearce, 1987). Once a response toward a stimulus has been learned, similar stimuli will elicit the same response. The more similar the stimulus is, the more likely the response will be. Thus, simply associating a response with an effect stimulus and assuming that this association is bi-directional will suffice to explain our data entirely.

A further important point to consider is whether participants did in fact learn an association between the action and the effect during training or rather an association between the location cue on the screen and the effect. Even though, we prefer the assumption that the perception of a similar effect directly selects the respective action toward that location, the observed facilitation of response times could have been mediated by a linkage between the location cues and the effect stimuli which then in the transfer phase led to a quicker perception of the learned stimulus location after perceiving the same or similar effects. Under this assumption, it is not necessarily true that action–effect learning is involved at all in our study and that the observed effects merely are the result of bi-directional S–S learning that leads to increased or decreased reaction times. The same results could have been observed, even if there never was any response during the training. With our design, this issue cannot be ruled out and only taking the current study by itself into account, simple S–S learning would be the simplest interpretation. However, we would like to point out that even if there was no response component in the training phase, i.e., there were only stimulus locations followed by certain other stimuli (i.e., the “effects”), action–effect learning could be conceivable. First, attending certain locations can be regarded as actions, second perceiving certain location can elicit covert motor responses that are typically associated with that location and thus lead to action–effect learning, even if there never was any observable response (Haider et al., 2018). Thus, a separation between perceptual and motor can sometimes be misleading (Hommel, 2019; Hommel et al., 2001) and action–effect learning might also be taking place in a more indirect way, by which the perceived stimulus location is associated with a certain effect stimulus and perceiving this effect stimulus elicits a motor response that is most fitting to the spatial information associated with that effect. Additionally, based on the high functional relevance and salience of motor responses, it seems unlikely that this crucial component of the task is not picked up by the cognitive system and that only S–S relations are learned instead. After all, the dorsal visual stream is assumed to connect visual (location) information with the motor system (e.g., Goodale, 2014). Possibly, future studies can rule this important issue with our current design out by creating designs that do not involve stimulus location cues or involve free choices.

Another limitation that needs to be addressed regards the choice of stimuli during the training and the test phase. First, the same stimuli appeared during training and during the test phase. Thus, we do not know whether the observed effects depend on the reappearance of the training stimuli during the test phase, in order for participants to draw the connection between the similar effects. Replications could repeat this design with entirely new stimuli during the test phase. Second, a further improvement could be to use a broader range of stimulus sets. If it can be shown that the transfer can be found for a broader range of stimuli, this could further improve the validity of the results and furthermore control possibly confounding factors that influence the perceives similarity (e.g., similar shape).

However, we would also like to point out that these first results, over two experiments that used different operationalizations of similarity, showed coherent results and are a promising start despite these limitations. Future studies can, thus, be more daring in using different stimuli and similarities. We do not know yet what exactly makes two stimuli similar. So, future research could focus on this question. Simple auditive stimuli might be a good starting point, more complex auditive similarity like tonal symmetry could be a following step. In the visual domain, it could be interesting to see whether our findings transfer to more complex visual effects. This could include an arbitrary response being associated with a more complex visual effect, like watching an avatar’s hand opening a bottle and later seeing an avatar turning the knob of an old radio with a similar movement.

A last important limitation of our study that should be noted, is that the overarching motivation behind our study was to investigate how humans flexibly use their acquired action–effect knowledge to pursue new goal that have never been achieved before. We did not show that the *anticipation* of new, similar effects or the intention to achieve them elicit a previously learned response. We have merely made a first attempt to start investigating this issue. In our study, participants saw stimuli and these stimuli affected response times, based on their perceived similarity to previously learned effect stimuli. We do not know what it was that participants anticipated or internally represented that drove this effect. Did the perception of the similar stimulus directly influence which responses to select or did the presentation of a similar stimulus active the representation of the learned effect? This should be clarified in future studies.

Lastly, future studies should also take a closer look at the mechanism behind this initial observation reported here. The need for internal models could further be investigated by whether a person learns to adjust their motor response based on the size and direction of the prediction error. The extent of an adjustment in the motor response might correspond to the extent of perceived similarity of the stimuli.

## Data Availability

The datasets generated during and/or analyzed during the current study are available from the corresponding author on reasonable request.

## Footnotes

1 Currently G*Power does not calculate sample sizes for within-subject interactions. For this reason, we calculated the sample sizes for the main effects and ran a sensitivity test after our data collection, to get an impression which interaction effect size can be observed given the sample size and power. For Experiment 1, this resulted in *η*_*p*_^*2*^ = .09 and *η*_*p*_^*2*^ = .08 for Experiment 2.
